# Hexavalent Chromium Immobilization in Soil by Sludge Biochar Tubules Under Fluctuating Groundwater: Performance, Mechanisms and Engineering Potential

**DOI:** 10.3390/molecules31173068

**Published:** 2026-08-31

**Authors:** Lin Chen, Yinger Deng, Yurou Hu

**Affiliations:** 1 Key Laboratory of Synergetic Control and Joint Remediation for Soil & Water Pollution, Ministry of Ecology and Environment, Chengdu University of Technology, Chengdu 610059, China; 2022010108@stu.cdut.edu.cn; 2 College of Environment and Civil Engineering, Chengdu University of Technology, Chengdu 610059, China; 2019010103@stu.cdut.edu.cn

**Keywords:** chromium-contaminated soil, sludge biochar, immobilization technique, groundwater table fluctuation

## Abstract

Although biochar has been widely applied for chromium (Cr)-contaminated soil remediation, its direct mixing with soil is limited by poor recoverability and insufficient long-term stability, while the role of groundwater table fluctuation (GTF) is often overlooked. In this study, we developed a biochar tubule-based strategy using Fe, Mg, and Al dual-modified sludge biochar (SBC) for Cr immobilization, and evaluated its performance, mechanisms, and engineering potential under GTF conditions. The demonstrated that modification substantially improved the microstructure and surface chemical properties of biochar. Experiments showed that Fe-SBC exhibited optimal immobilization performance, followed by Mg-SBC, Al-SBC, and SBC. Compared with the controls, Fe-SBC reduced Cr release by 97.66% (slightly contaminated) and 71.40% (heavily contaminated), while the corresponding Cr-retained amounts (predominantly in stable fractions) increased by 155.68% and 257.42%; meanwhile, 7.05% and 9.05% of the total amounts were removed by the biochar tubule, respectively. Mechanistic analysis revealed that Cr(VI) was transported into the biochar tubules via tubule-induced preferential flow and subsequently immobilized through synergistic adsorption, reduction, complexation, and precipitation processes, with redox reactions playing a predominant role (67.37–98.87%). Additionally, the environmental risks, application cost and prospects of biochar were evaluated. This study provides new insights into sludge resource utilization and soil immobilization techniques.

## 1. Introduction

Cr, a ubiquitous environmental heavy metal (HM), primarily exists as trivalent Cr(III) and Cr(VI). Compared with the relatively stable and less toxic Cr(III), Cr(VI) is highly soluble, mobile, and toxic, typically occurring as oxyanions, such as ${\mathrm{HCrO}}_{4}^{-}$, ${\mathrm{CrO}}_{4}^{2-}$, and Cr_2_$O_{7}^{2-}$. These properties facilitate its mobility in soil and groundwater, posing significant ecological risks [1,2]. Notably, the redox transformation between Cr(III) and Cr(VI) is highly sensitive to environmental conditions. Groundwater table fluctuation (GTF), a common hydrological process, can significantly alter the redox status to influence Cr speciation and mobility [3]. For example, a rising water table introduces oxygen (O_2_) into previously anoxic zones, promoting the formation of reactive oxygen species (H_2_O_2_ and ·OH) that facilitate the oxidation of Cr(III) to Cr(VI) [4]. Furthermore, GTF reduces vadose zone thickness, shortens migration pathways, and increases the risk of groundwater pollution [5]. Despite these critical influences, GTF has rarely been considered in studies of Cr immobilization in soils.

Biochar has been widely applied for the remediation of Cr-contaminated soils due to its low cost and abundant feedstocks. Among various feedstocks, municipal sludge represents an abundant waste resource, with an annual production of approximately 90 million tons in China [6]. Converting sludge into biochar offers a sustainable pathway for waste valorization and pollution control. However, pristine sludge biochar (SBC) generally exhibits better adsorption toward cationic HMs, whereas its performance in capturing anionic Cr(VI) (chromate and dichromate) species is relatively limited. Metal salt modification has been demonstrated as an effective strategy to improve SBC performance, particularly using Fe, Mg, and Al species that are naturally abundant in soils. Furthermore, Shen et al. [7] reported that loading Fe(III)-loaded SBC significantly enhances Cr(VI) removal, and the performance positively correlated to metal loading [8]. This suggests that improving the pore structure to enable higher metal loading is key to enhancing Cr(VI) immobilization. Co-pyrolysis of sludge with biomass provides a simple method to reduce sludge hazards while increasing the porosity and specific surface area (S_BET_) of biochar. Therefore, combining the dual-modification strategies is crucial for addressing the limitations of sludge pyrolysis alone.

Another critical limitation in current remediation strategies lies in the application mode of biochar. Conventional approaches rely on the direct mixing of biochar with soil, which not only hinders material recovery and reuse [9,10] but also raises concerns regarding long-term stability due to aging, microbial activity, and potential secondary pollution [11,12,13]. These challenges highlight the urgent need for separable and retrievable biochar-based systems (biochar tubule) for the sustainable remediation and long-term safe management of Cr-contaminated soils.

In this study, biochar tubules were developed for Cr(VI) immobilization in soil under GTF conditions. Modified biochars were synthesized via a dual-modification strategy combining metal salt activation (FeCl_3_, AlCl_3_, and MgCl_2_) and co-pyrolysis with walnut shell, and their performances in immobilizing Cr(VI) were systematically evaluated. The objectives of this study are to: (1) characterize the physicochemical properties of modified biochars and clarify their roles in Cr(VI) immobilization; (2) investigate the immobilization behaviors of different biochars on Cr(VI) under GTF conditions and elucidate the underlying mechanisms; and (3) evaluate the engineering applicability of biochar tubule. This study provides new insights into the application of sludge-derived biochar materials for Cr-contaminated soil remediation under dynamic hydrological conditions.

## 2. Results and Discussion

### 2.1. Characteristics of Different Biochars

The physicochemical properties and elemental compositions of different biochars are summarized in Tables S1 and S2. Modification substantially altered the surface properties of the SBC. Fe/Al-SBC exhibited acidic characteristics, whereas Mg-SBC was alkaline, highlighting the strong influence of different modifiers on biochar surface chemistry. Meanwhile, modification increased the S_BET_ and reduced the average pore diameter, while the pore volume increased only for Mg-SBC. The lack of pore volume enhancement in Fe-SBC and Al-SBC may result from partial pore blockage by Fe- and Al-containing species [14]. Mg-SBC showed the most pronounced pore structure improvement, possibly due to MgCl_2_-induced decomposition of volatile components during pyrolysis [15]. Moreover, the incorporation of walnut shells diluted the intrinsic HM contents of the biochar, reducing the potential environmental risk. Elemental analysis revealed increased C and O contents in the modified biochars, potentially contributing to their Cr(VI) adsorption and immobilization performance [16].

**Figure 1:**
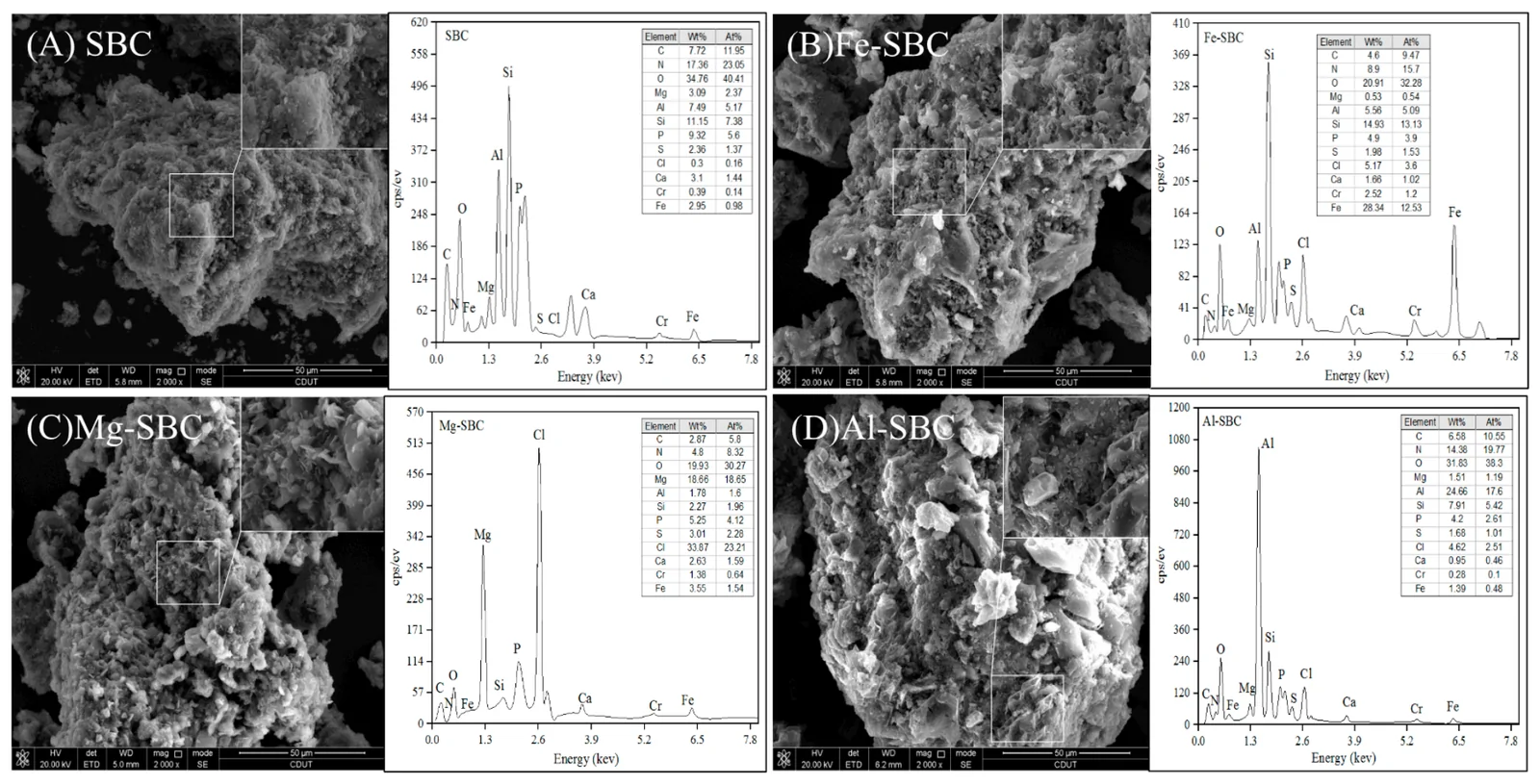
SEM images and corresponding EDS elemental maps (**A**–**D**).

The SEM-EDS images (Figure 1A–D) show the surface morphology and element distribution (semi-quantitative) of the biochars, with detailed images provided in Figure S1. Compared with the relatively smooth surface of the SBC, the modified biochars exhibited rougher surfaces with abundant cracks and pores. Fe-SBC exhibited a relatively uniform macroporous structure, whereas Mg-SBC and Al-SBC displayed fragmented, irregular porous structures and frameworks. These structural features provide increased binding sites for Cr [17], consistent with the enhanced S_BET_ and total pore volume (Table S1). EDS mapping further confirmed the homogeneous incorporation of Fe, Mg, and Al into the biochar matrix, with their mass fractions increasing by 441.87%, 1380.95%, and 381.64%, respectively.

**Figure 2:**
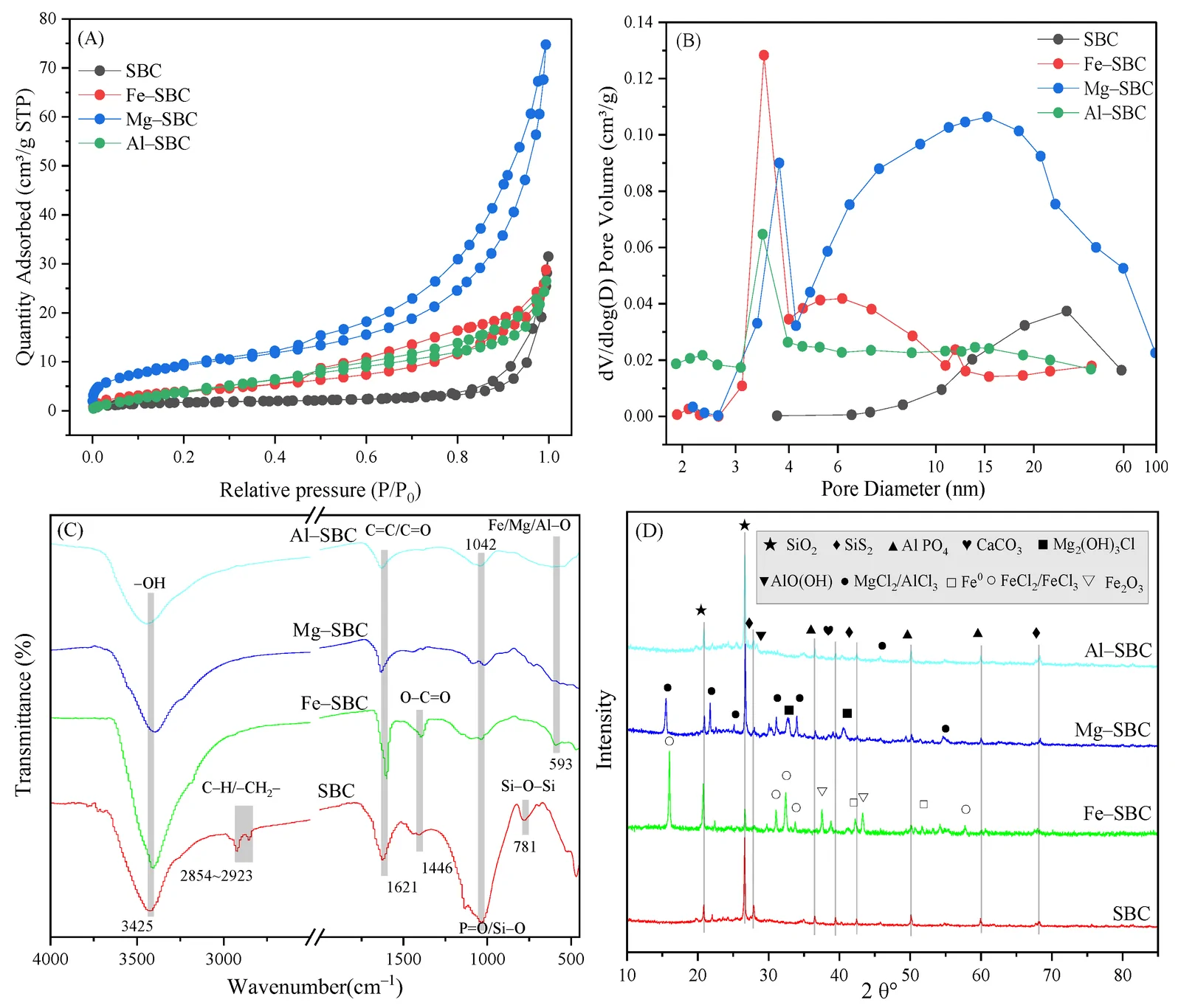
N_2_ adsorption–desorption isotherms (**A**); and pore size distribution curves (**B**); FTIR (**C**) and XRD (**D**) patterns of biochars.

Figure 2A,B illustrate the pore characteristics of the biochars. The modified biochars exhibited pronounced slopes and clear hysteresis loops in the N_2_ adsorption–desorption isotherms at P/P_0_ > 0.4, suggesting the presence of mesoporous structures in the biochars, whereas the SBC showed a sharp adsorption increase at high relative pressures, characteristic of macroporous structures (Figure 2A) [18]. Pore size distribution further revealed that the modified biochars exhibited a broader pore diameter distribution and a shift toward smaller pores (2–4 nm), particularly for Fe-SBC, accompanied by a reduced average pore diameter.

The FTIR spectra (Figure 2C) showed oxygen-containing functional groups, including -OH, C=C/C=O, and C-O/Si-O groups. After modification, the C-H, -CH_2_-, and Si-O-Si bands disappeared or weakened, accompanied by the appearance of new metal–oxygen vibration bands, indicating the biochars’ structural changes and the incorporation of Fe, Mg, and Al species. The XRD patterns (Figure 2D) further showed the formation of metal-containing crystalline phases after modification, supporting the successful modification of the biochars, consistent with the EDS result. These changes indicate that metal modification altered the surface chemical environment and mineral composition of the biochars, which may provide additional active sites for Cr(VI) immobilization.

Table 1 compares the adsorption capacities of different biochars toward Cr(VI) and Cr(III). Under comparable experimental conditions, Fe modification markedly enhanced Cr(VI) adsorption, with the high performance of the ZVI-modified biochars potentially attributed to their stronger reducing capacity. The effects of the feedstock type (sludge and sugarcane residue) on Cr(VI) adsorption were relatively limited. Additionally, Mg-SBC substantially improved Cr(III) adsorption than other SBCs, and Cr(III) adsorption varied with the feedstock type and pyrolysis conditions.

### 2.2. Effects of Hydrological Conditions on Immobilization Performance

**Figure 3:**
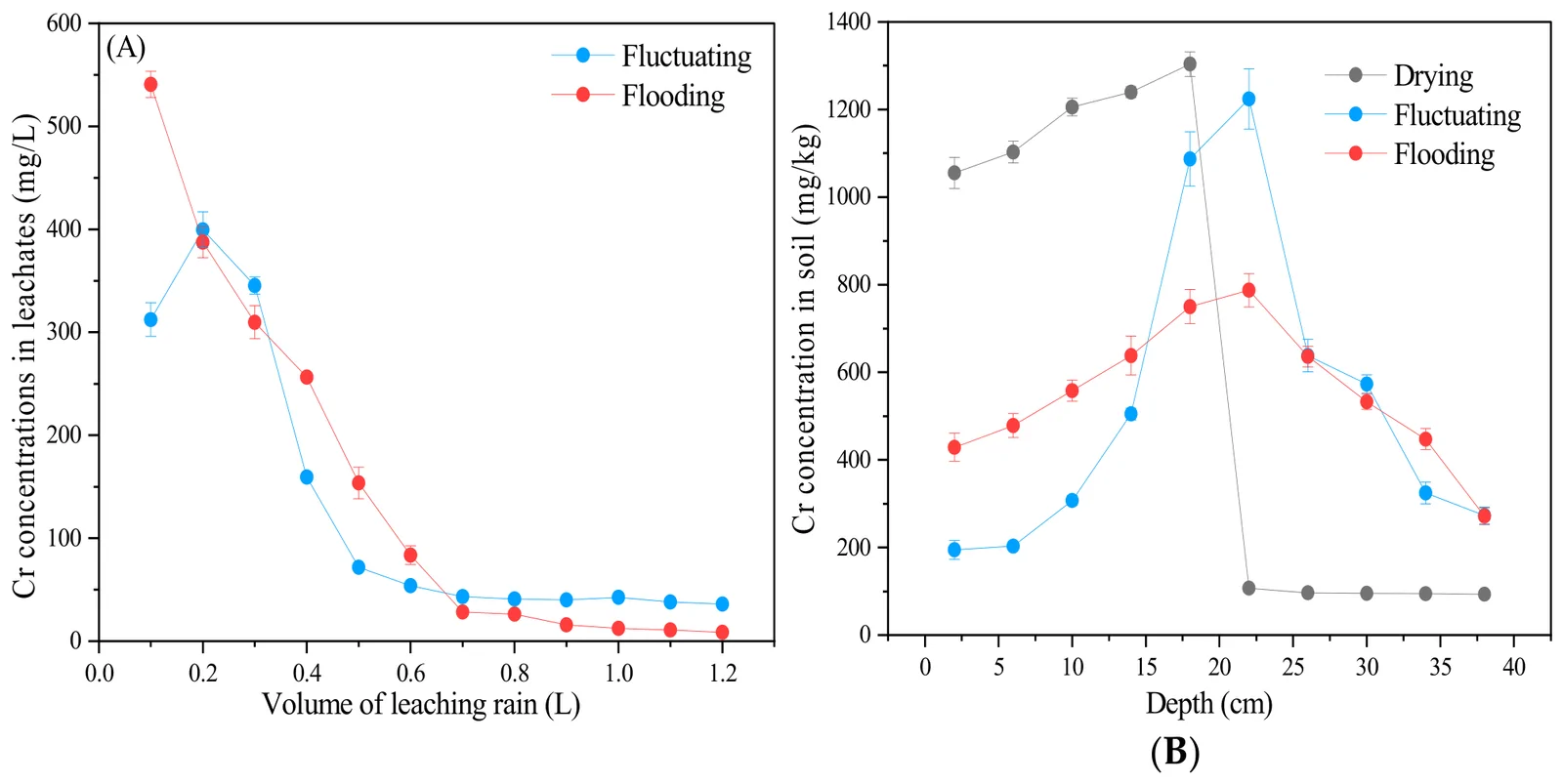
Cr leaching behavior (**A**) and profile distribution (**B**) under different hydrological conditions. Note: Initial soil Cr content > 1300 mg/kg; Fe-SBC was applied as the amendment.

Figure 3 illustrates the variations in Cr concentrations in the leachates and soil profiles under drying, flooding, and fluctuating groundwater conditions. The results show that under fluctuating conditions, the Cr concentrations in the leachate initially increased and subsequently declined, whereas a continuous decline was observed under flooded conditions (Figure 3A). This difference is mainly attributed to distinct soil–water dynamics. During groundwater drawdown, transient negative pore water pressures may enhance the downward mobilization of labile Cr fractions by altering the soil–water dynamics [25]. In contrast, sustained saturation under flooded conditions facilitates the persistent dissolution and leaching of soluble Cr. The lower concentration peak of Cr observed under fluctuating conditions may result from intermittent wetting, which limits pore water saturation to constrain Cr dissolution and transport.

In the soil profile, limited migration of Cr occurred under drying conditions, whereas considerable transport occurred under flooded and fluctuating conditions (Figure 3B), highlighting water’s key role in Cr mobility. Flooding resulted in a relatively uniform Cr distribution, while fluctuating conditions caused pronounced accumulation at 18–22 cm. Similar accumulation zones have been observed during HM leaching and were attributed to the reduction in the size and number of seepage pathways that progressively restricted further contaminant migration [26]. Furthermore, O_2_ ingress during wetting–drying cycles may promote Fe(II) oxidation to Fe(III) and enhance Fe-Cr complex formation, thereby potentially increasing Cr retention in deeper layers [4]. These results emphasize the interplay between hydrodynamics and redox-driven transformations in controlling Cr migration and immobilization.

### 2.3. Analysis of Immobilization Behavior

#### 2.3.1. Leaching Concentrations and Amounts

**Figure 4:**
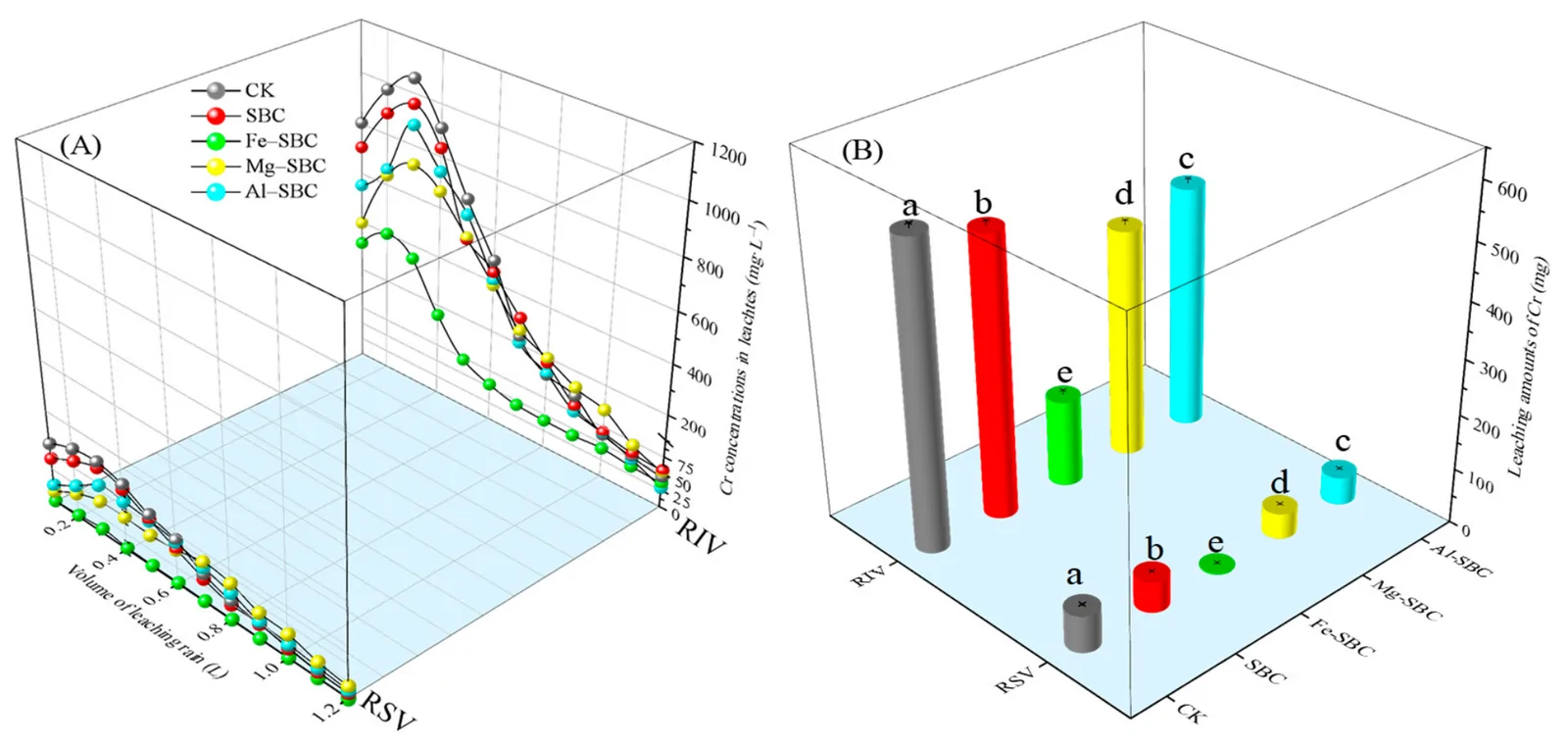
Variations in Cr concentrations with leaching volume (**A**); cumulative leaching amounts (**B**). CK: quartz sand replaces biochar. Lowercase letters (a–e) in (**B**) indicate statistically significant differences among treatments (*p* < 0.05).

As shown in Figure 4A,B, all biochar treatments significantly reduced both the leaching concentration and cumulative release of Cr during the GTF process. Specifically, the Cr leaching concentration initially increased and then decreased with the increasing cumulative leachate volume, suggesting that the readily mobilized Cr was gradually depleted or immobilized during the GTF cycles. Among them, Fe-SBC exhibited the highest efficiency, lowering the peak Cr concentration in the RSV (250 mg/kg, risk screening values) and RIV (1300 mg/kg, risk intervention values) soils from 116.53 mg/L to 6.11 mg/L (a 94.76% reduction) and from 1056.82 mg/L to 345.37 mg/L (a 67.32% reduction), respectively, compared with CK. Mg-SBC induced a relatively stable leaching peak in the RSV soil, indicating effective retardation of Cr released, which may be partly associated with its relatively wide pore size distribution and higher S_BET_ compared with the other biochars (Figure 2B and Table S1).

Cumulative Cr release was significantly lower (*p* < 0.05) in all biochar treatments, with Fe-SBC again showing the greatest reduction, followed by Mg-SBC, Al-SBC, and SBC (Figure 4B). Specifically, Fe-SBC decreased the total Cr leaching from 67.04 mg to 1.57 mg in the RSV soil (97.66% reduction) and from 553.49 mg to 158.31 mg in the RIV soil (71.40% reduction) relative to CK. Overall, Fe-SBC outperformed all other treatments in minimizing both the peak leaching concentrations and cumulative Cr release. This superior performance may be attributed to its relatively high pore volume in the 4–5 nm pore diameter range (Figure 2B), which favors Cr(VI) adsorption, together with Fe-based functional groups that promote complexation and the reduction of Cr(VI) to Cr(III) by reductive Fe species, followed by precipitation under alkaline soil conditions [4,27].

#### 2.3.2. Vertical Migration of Total Cr and Labile Cr Fractions in Soil Columns

**Figure 5:**
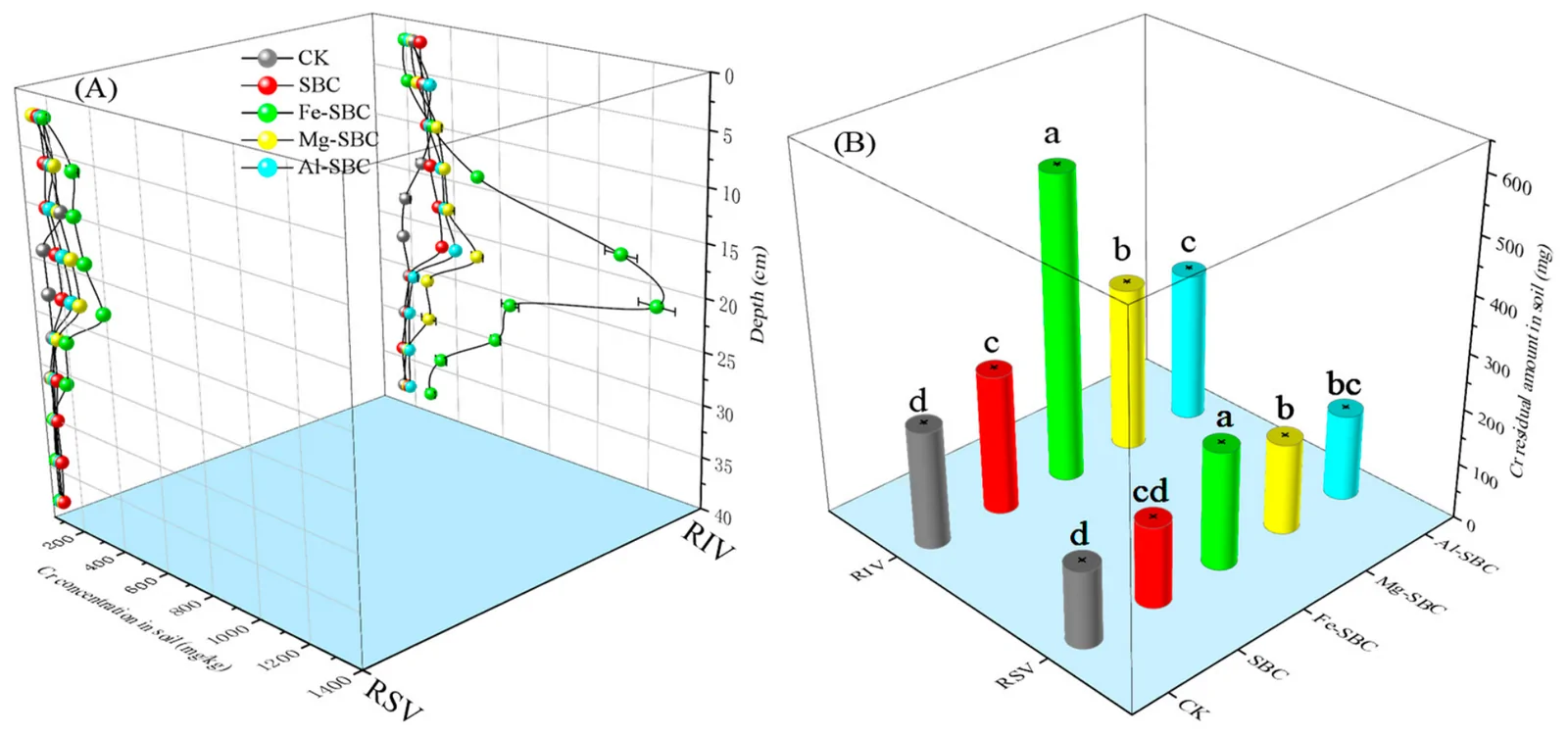

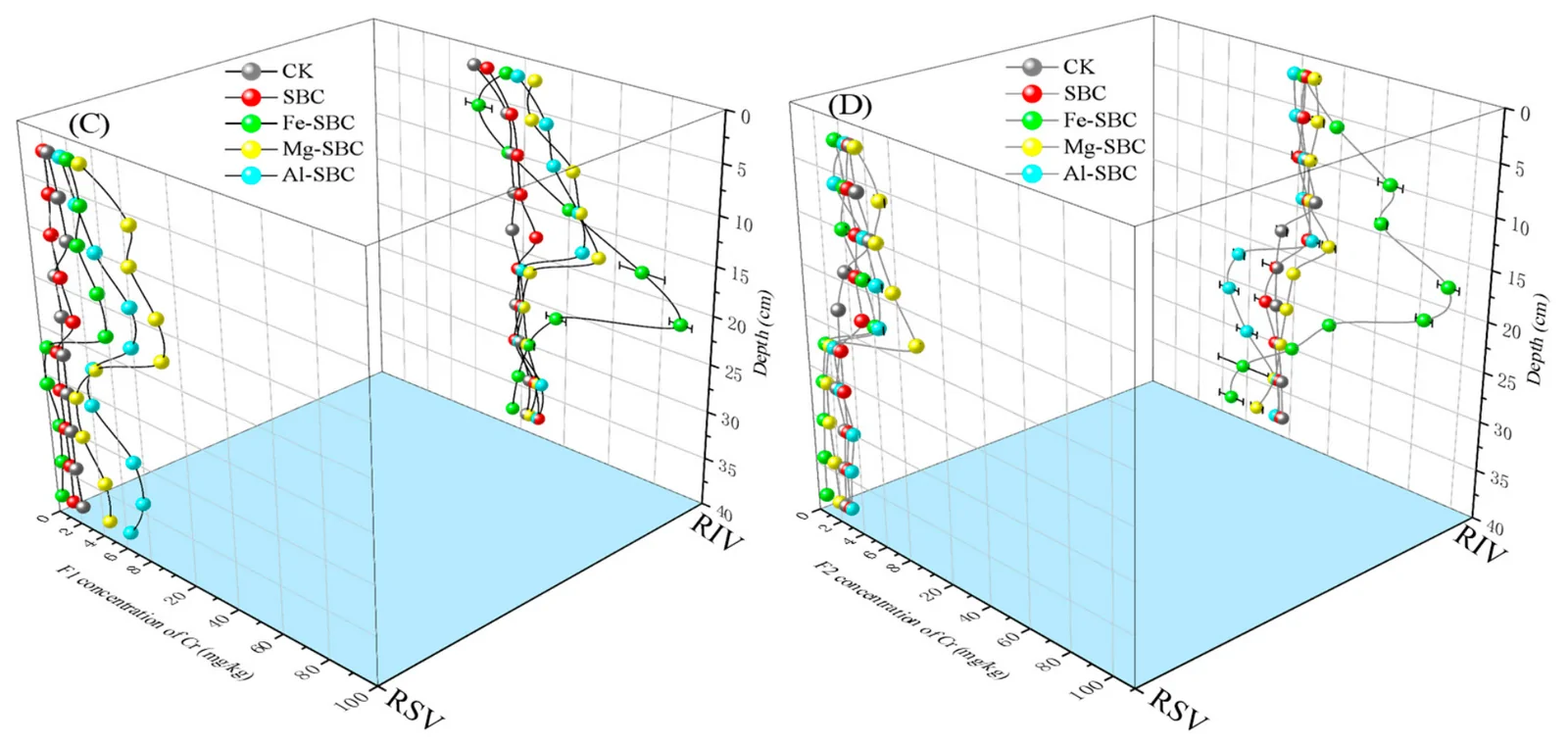
Vertical distribution of total Cr concentrations in the soil profile (**A**), cumulative residual total Cr in the soil columns (**B**), and vertical distribution of F1 (**C**) and F2 (**D**) fractions. Lowercase letters (a–d) in (**B**) indicate statistically significant differences among treatments (*p* < 0.05).

Figure 5A shows the vertical distribution of total Cr after the fluctuation process. In both contaminated soils, the Cr concentrations in the biochar treatments gradually increased with depth, and reached a maximum near the interface between the contaminated and clean soil layers, followed by a sharp decline and stabilization. In the RIV soil, Fe-SBC and the SBC exhibited the highest and lowest Cr accumulation peaks, respectively, corresponding to 7.53- and 2.10-fold increases compared with the CK group. The accumulation of Cr within the contaminated layer indicates effective immobilization by the biochar, consistent with previous reports [28,29]. In contrast, the Cr concentrations in CK continuously decreased below 10 cm, further confirming the role of the biochar in restricting downward Cr migration. Notably, the peak position (RIV) at approximately 22 cm suggests slight downward redistribution of Cr (Figure 5A), which may be associated with the remobilization and subsequent transport of weakly bound Cr under cyclic hydraulic fluctuations [30]. As shown in Figure 5B, all biochar treatments significantly increased Cr retention across the soil profile (*p* < 0.05). Fe-SBC exhibited the strongest effect, with Cr retention in the RSV and RIV soils increasing by 155.68% and 257.42%, respectively, compared with CK. No significant differences were observed between the Al-SBC and SBC treatments, which may be partly related to the low solubility of Al species and their limited contribution to Cr immobilization under the experimental conditions.

To further evaluate Cr immobilization and passivation, the vertical distributions of labile Cr fractions (F1(exchangeable fraction) and F2 (carbonate-bound fraction)) were analyzed (Figure 5C,D). Except for CK, both fractions showed similar vertical patterns to the total Cr, with the maximum concentrations occurring near 20 cm depth. In the RSV soil, Fe-SBC significantly reduced the F1 and F2 fractions compared with Mg-SBC and Al-SBC, in contrast to the total Cr distribution, indicating enhanced conversion of labile Cr into more stable forms. This effect was mainly attributed to the reduction of Cr(VI) by Fe(0) and Fe(II) species on Fe-SBC, followed by Cr(III) precipitation as Cr(OH)_3_ [31,32]. However, in the RIV soil, Fe-SBC exhibited relatively higher F1 and F2 concentrations. The F1 peak decreased from 7.53-fold to 4.66-fold relative to the total Cr peak, representing a greater reduction than that observed for the SBC (from 1.84-fold to 1.72-fold), indicating more effective passivation after modification; the remaining high F1 levels suggest that limited reducing agents were consumed by excessive Cr(VI). Consequently, some Cr(VI) may have remained as Fe-O-Cr(VI) complexes in the soil [7], resulting in elevated F1 and F2 fractions. Similar observations were reported by Nguyen et al. [33], who found that FeCl_3_-modified biochar exhibited superior Cr immobilization than AlCl_3_- or CaCl_2_-modified biochar due to the formation of Fe-O-based specific complexes.

#### 2.3.3. Distribution of Cr Speciation in Soil

**Figure 6:**
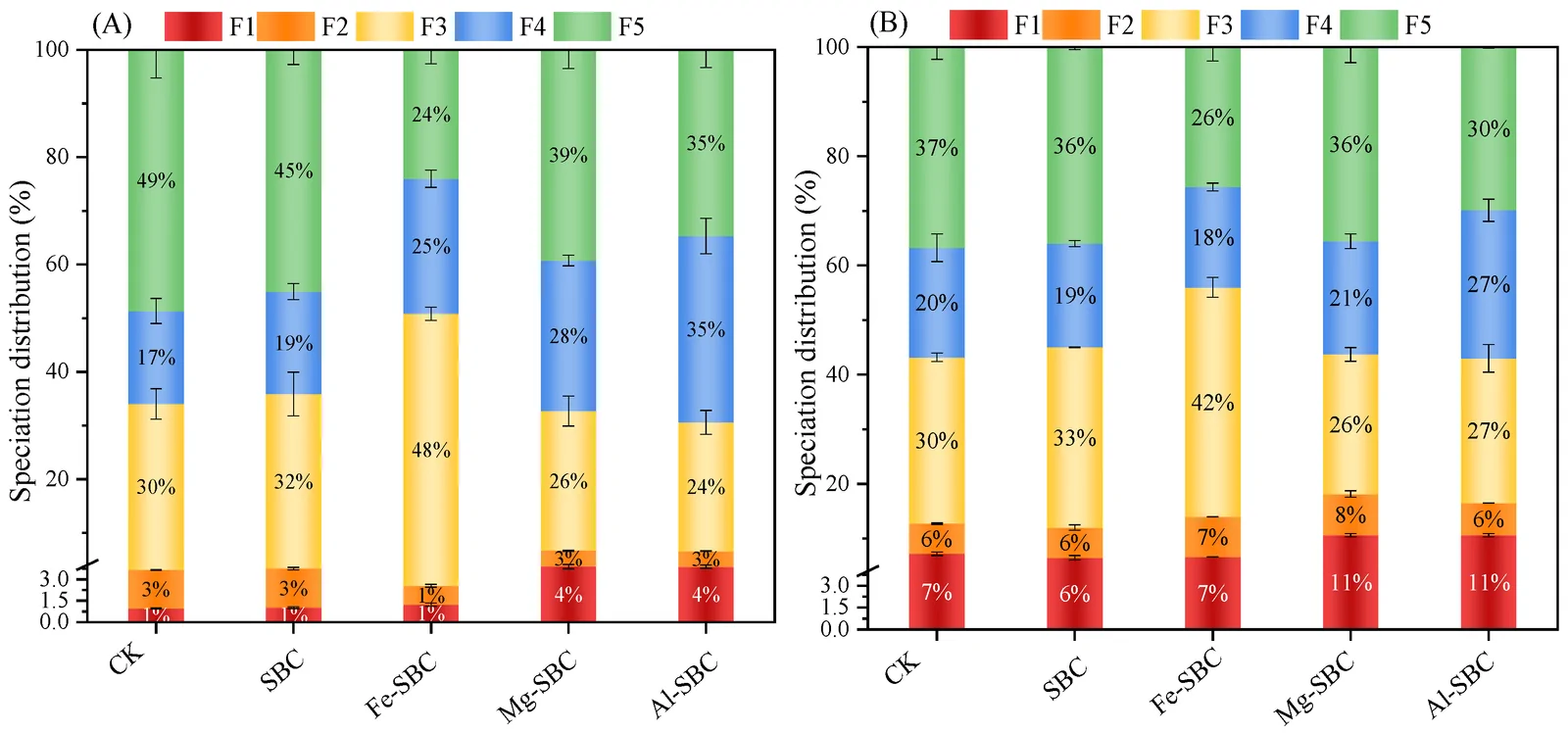
Cr speciation distribution in soil. RSV soil (**A**), RIV soil (**B**).

Figure 6 shows the distribution of Cr speciation in soils after biochar treatments. The results indicate that Cr predominantly existed as F3, F4, and F5 fractions (F3, F4, and F5 represent the Fe-Mn oxide-bound fraction, organic-bound fraction, and residual fraction, respectively), accounting for 93.31–97.46% (RSV) and 81.86–88.02% (RIV) of the total Cr in soils, indicating residual Cr in soil was mainly present in relatively stable fractions with low migration risk. Different biochars induced distinct transformations of Cr speciation. Fe-SBC significantly increased the proportion of F3 (48.27% and 42.00%), which may be attributed to the adsorption, complexation, and co-precipitation of Cr by Fe oxides. In contrast, alkaline Mg-SBC (Table S1) promoted the transformation of Cr toward the F5 fraction (39.25% and 35.56%), which was likely associated with the increase in soil pH and the subsequent precipitation of Cr as hydroxides or carbonates [34]. In the RSV soil, Fe-SBC significantly reduced the proportion of F1 + F2 fractions compared with Mg/Al-SBC. Combined with the total residual amounts in the soil (Figure 5B), it indicated Fe-SBC not only enhanced Cr immobilization in the soil but also promoted the transformation of Cr(VI) into more stable fractions. However, the proportion of F1 + F2 fractions was overall higher in the RIV soil than in the RSV soil, which may be related to the higher Cr(VI) contamination level in the RIV soil [35].

### 2.4. Cr Mass Balance and Partitioning in Soil Columns

**Figure 7:**
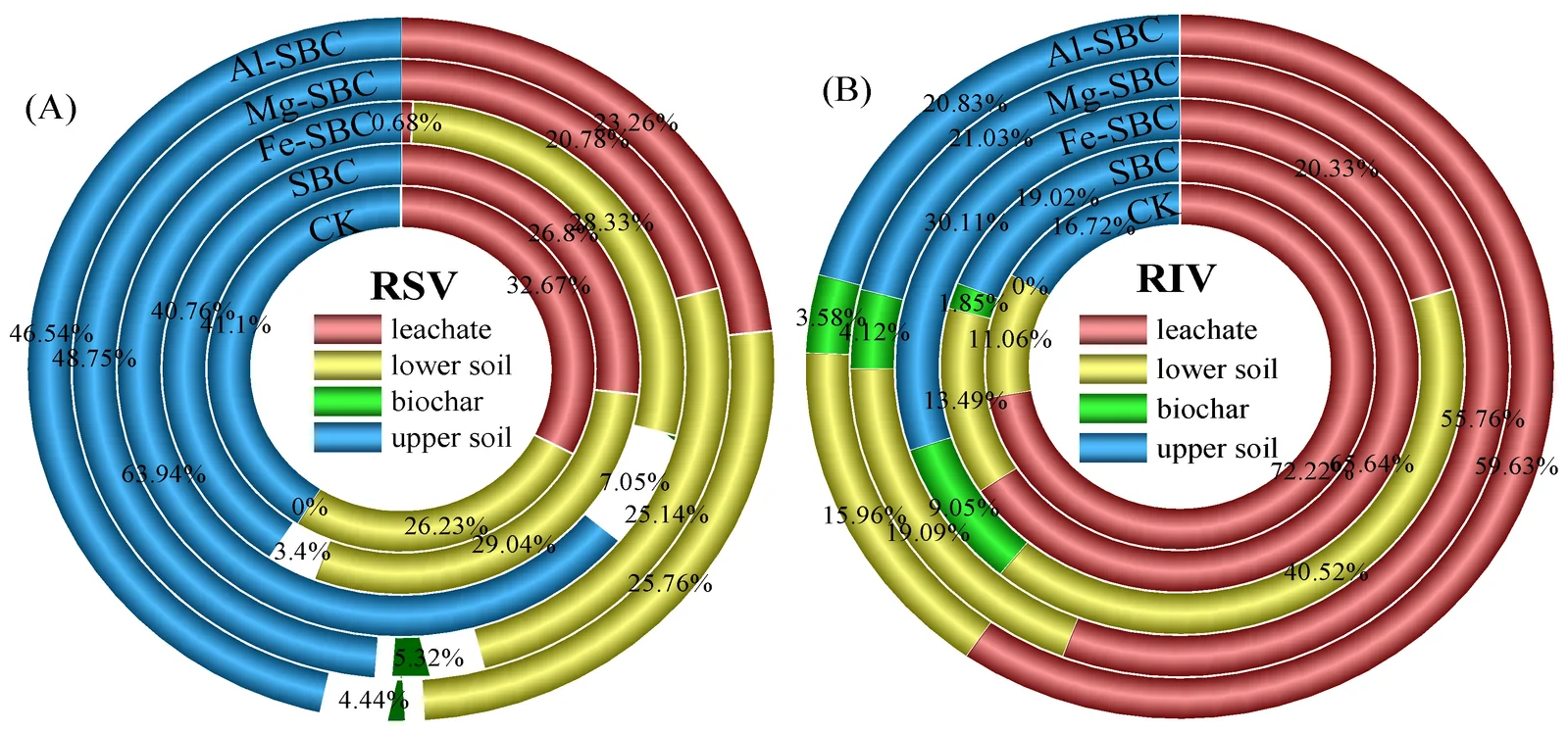
Percentage distribution of Cr mass among different components of the soil columns. RSV soil (**A**), RIV soil (**B**).

The Cr mass balance before and after the experiments was analyzed to quantify Cr distribution among different components (Table 2 and Figure 7). The results showed that the mass balance errors (Equation (2)) ranged from 0.90% to 8.28%. The errors of most treatments were below or around 5%, indicating reasonable closure between the initial and final Cr masses. The maximum error was observed for Fe-SBC in the RSV soil, which may be related to its stronger Cr immobilization performance than the other biochars. This effect may be more pronounced in the RSV soil with a lower initial Cr concentration (Figure 7A), where Fe-SBC may promote more heterogeneous Cr redistribution under GTF conditions, resulting in cumulative errors during soil sampling, analysis, and calculation.

(1) $$\delta_{M}=\left|\frac{\sum T_{a}-\sum T_{b}}{\sum T_{b}}\right|\;\times \;100\%$$

where $\delta_{M}$ represents the mass balance error, %; $T_{a}$ and $T_{b}$ represent the Cr mass before and after the experiment, respectively (mg).

The results of mass balance showed that the biochar treatment significantly increased Cr retention in the upper soil (0–20 cm), accompanied by a reduction in Cr mass in the leachate (Table 2). Compared with CK, Fe-SBC increased Cr retention in the upper soil by 55.57% and 80.08% in the RSV and RIV soils, respectively, while reducing Cr leaching by 97.92% and 71.85%. These results demonstrate that biochar tubules effectively restricted Cr migration through enhanced retention in soil and reduced leaching losses. The corresponding percentage distributions in Figure 7 further show that up to 7.05% and 9.05% of the total Cr were captured by the biochar in the RSV and RIV soils, respectively, which could be physically separated and removed from the soil system, highlighting the potential of biochar tubules for Cr remediation through an “adsorption–separation–removal” strategy.

### 2.5. Mechanisms of Cr Transport and Immobilization Regulated by Biochar Tubules

#### 2.5.1. Transport-Controlled Mechanism

The results of this study show that the insertion of biochar tubules induced pronounced heterogeneity in Cr distribution within the contaminated soil layer (i.e., upper soil), with a 6.28-fold difference between the upper and lower contaminated soil layers (Section 2.3.2). In contrast, previous studies using conventional uniform biochar–soil mixing have reported relatively limited concentration variation in the soil profile of Cd within the contaminated soil layer [28]. This difference may be attributed to the distinct spatial distribution of biochar and the resulting flow conditions under the two remediation modes. In the uniform mixing mode, biochar is evenly distributed throughout the soil matrix, resulting in relatively homogeneous Cr adsorption and immobilization. In contrast, in this study, the biochar tubules exist in a discrete configuration, creating localized high-permeability zones that induce preferential flow pathways under hydraulic forces. As a result, Cr-laden leachate is preferentially directed into the biochar tubules, where Cr is efficiently adsorbed and immobilized, thereby reducing the Cr concentrations in the upper portion of the 0–20 cm soil layer. With increasing depth within this soil layer, the effect of preferential flow gradually decreases as the hydraulic gradients weaken, while the chemical immobilization by the biochar becomes more pronounced, promoting Cr accumulation in the lower portion of the 0–20 cm soil layer. Additionally, weakly bound Fe, Mg, and Al species on the biochar surface may desorb into the surrounding soil under dynamic hydrodynamic conditions, subsequently migrating and accumulating in deeper layers. These released metal species can complex with dissolved Cr, further enhancing its immobilization [36]. ChenDai [9] further confirmed that modificatory Fe species released from biochar can form Fe–O–Cr complexes in soil, highlighting the critical role of free Fe species in Cr immobilization. After the experiment, the increased concentrations of Fe, Mg, and Al in the soil confirm that the element loaded on the biochar migrated into the soil under GTF conditions (Table S3).

#### 2.5.2. Cr Immobilization Mechanisms by Different Biochars

**Figure 8:**
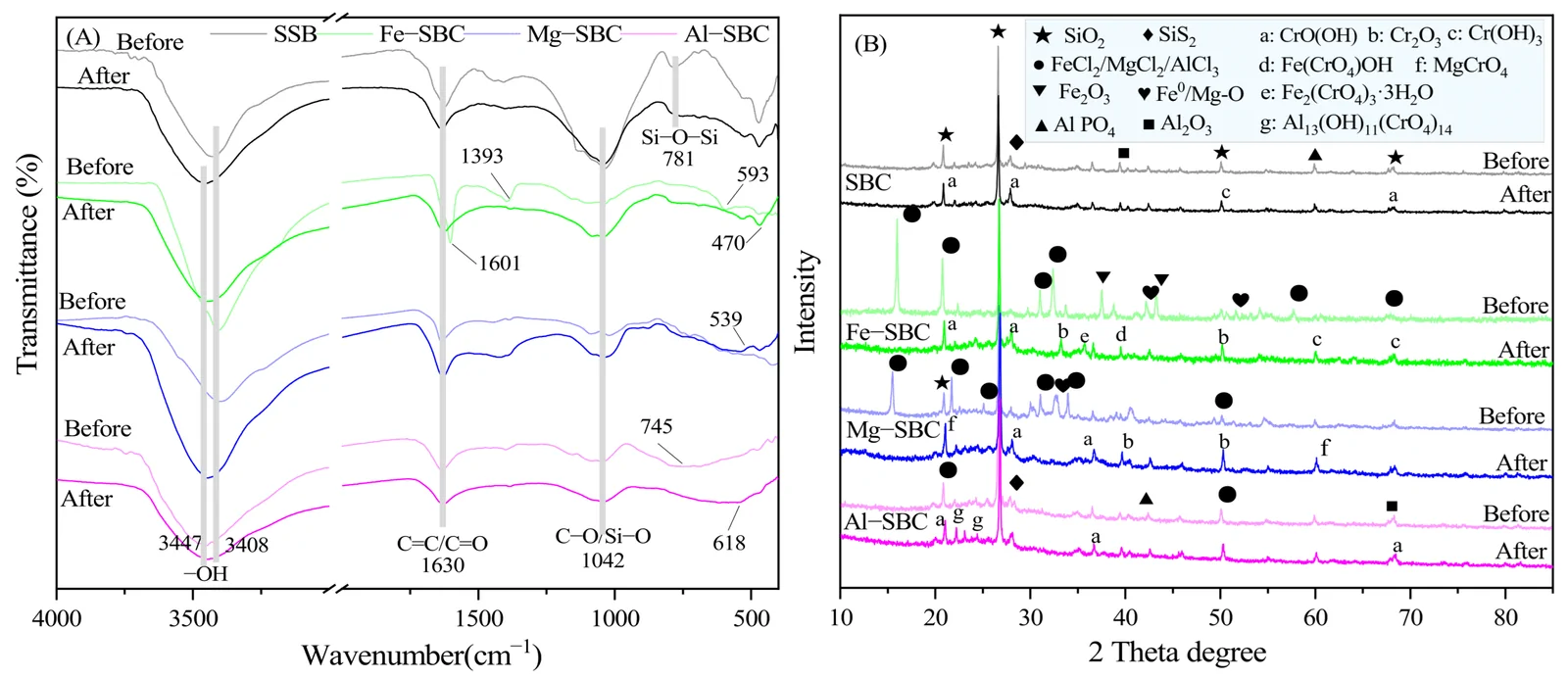

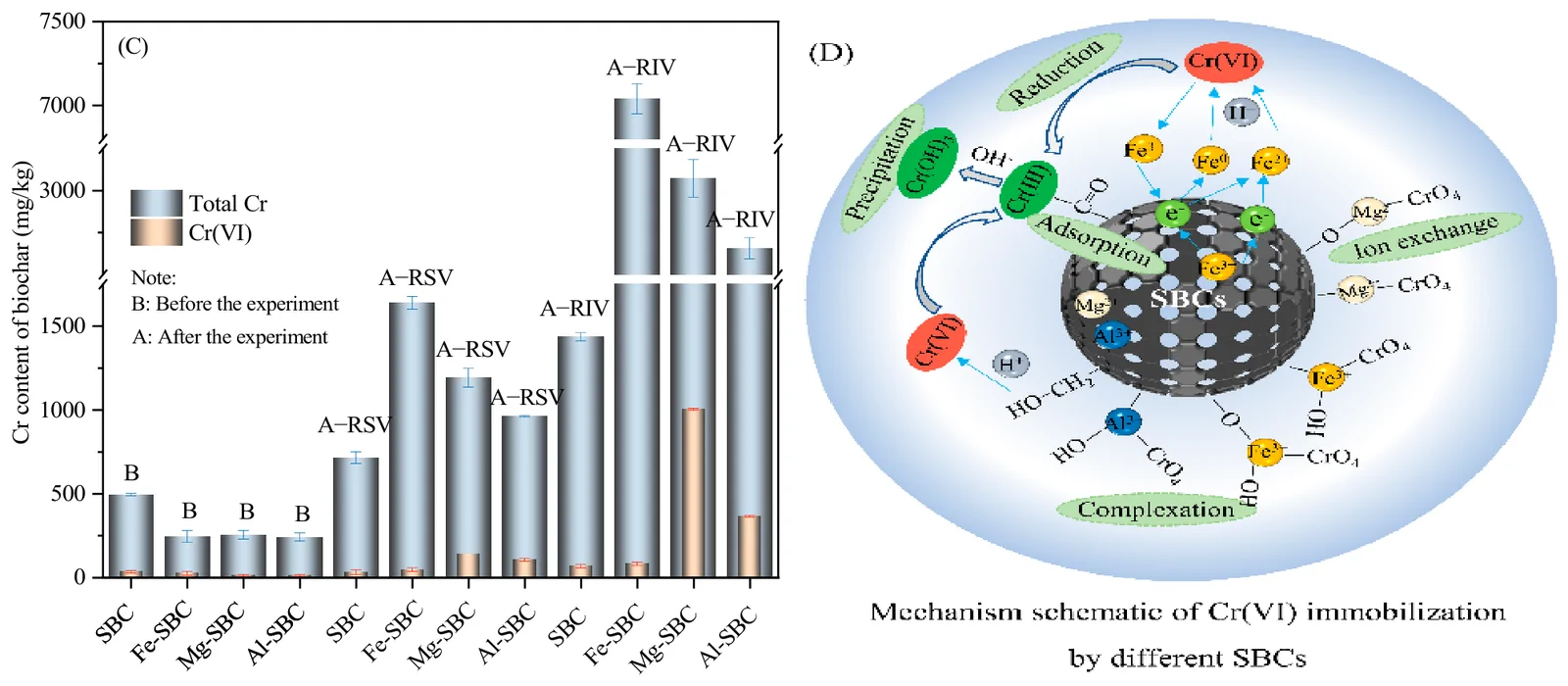
FTIR spectra (**A**) and XRD patterns (**B**) of biochars before and after experiment; total Cr and Cr(VI) amounts (**C**) in biochars; schematic illustration of Cr immobilization mechanisms (**D**).

The presence of Cr on the biochar surfaces after the experiment was confirmed by EDS analysis (Figure S2). To further elucidate the immobilization mechanisms, FTIR spectroscopy was employed to characterize the functional groups of the biochars (Figure 8A). After the experiment, the -OH peaks of the four biochars shifted to 3447 cm^−1^, indicating their involvement in Cr(VI) reduction (Equation (3)) [37]. Furthermore, changes in metal–oxygen bond vibrations further supported their role in immobilization. In Fe-SBC, the peak at 1601 cm^−1^ (associated with C=O groups [38]) shifted and weakened, while the peak at 1393 cm^−1^ (associated with COOH groups [39]) disappeared, implying that these functional groups participated in the reduction and adsorption of Cr (Equations (4) and (5)) [40]. After the experiment, the XRD analysis results show that CrO(OH) (PDF # 70-1115) was detected in the four biochars, indicating Cr(VI) reduction followed by precipitation as Cr(III), representing a dominant immobilization pathway. Distinct crystalline phases were observed among different biochars. In Fe-SBC, the attenuation or disappearance of FeCl_2_ and FeCl_3_ peaks indicates the involvement of Fe(II)/Fe(III) in Cr(VI) reduction and complexation. The formation of Cr_2_O_3_ (PDF # 25-1437) and Cr(OH)_3_ (PDF # 16-0817) further supports Cr(VI) reduction, while the Fe_2_(CrO_4_)_3_·3H_2_O (PDF # 31-0620) and Fe(CrO_4_)OH (PDF # 20-0511) suggests the formation of Fe–Cr-containing phases, potentially through complexation or coprecipitation. These results demonstrate synergistic Cr(VI) immobilization via reduction, complexation, and coprecipitation in Fe-SBC. In contrast, MgCrO_4_ (PDF # 23-0383) and Al_13_(OH)_11_(CrO_4_)_14_ (PDF # 32-0008) detected in Mg-SBC and Al-SBC suggest Cr(VI) immobilization via ion exchange and complexation.

(2) $$2{{Cr}_{2}O}_{7}^{2-}+3CH_{2}-OH+{16H}^{+}\to 4{Cr}^{3+}+11H_{2}O+3COOH$$

(3) $${{Cr}_{2}O}_{7}^{2-}+R-C=O\to R-C=O\ldots HOCrO_{3}^{-}$$

(4) $${Cr}^{3+}+3R-COOH\to {3H}^{+}+{\left(R-COO\right)}_{3}Cr$$

The amounts of Cr(VI) immobilized by different biochars are presented in Figure 8C. Overall, most Cr(VI) was reduced to Cr(III), indicating that reduction was the dominant mechanism (67.37–98.87%) in Cr(VI) immobilization. In the RIV soil, higher residual Cr(VI) was observed in Mg-SBC and Al-SBC, accounting for 32.63% and 13.66%, respectively, whereas Fe-SBC contained only 1.13%. This result suggests that Fe modification substantially enhanced the electron transfer capability of the biochar and promoted Cr(VI) reduction. The superior reduction performance of Fe-SBC can be attributed to the Fe(III)/Fe(II)/Fe(0) redox cycle, where Fe(II) and Fe(0) act as electron donors to reduce Cr(VI) to Cr(III), while biochar facilitates the electron transfer and regeneration of reduced Fe species [41,42]. Hu et al. [43] and KeRen [32] also demonstrated that the Fe-modified biochar enhanced Cr(VI) removal through Fe-mediated redox reactions. Based on these results, the proposed mechanisms of Cr immobilization are illustrated in Figure 8D.

### 2.6. Environmental and Engineering Implications

#### 2.6.1. Environmental Risk and Recyclability of Biochar

Considering the coexistence of other HMs (Cu, Pb, Cd, Zn, and Ni) in sludge, their concentration changes were also monitored to assess potential leaching risks (Table 3). The results showed that Cd, Ni, and Zn exhibited relatively higher release from the SBC, whereas their release was effectively suppressed by the modified biochars. The risk assessment results (Table S4) indicated overall low ecological risk, although Cd in the SBC reached a moderate level, which decreased to low after the modified biochar treatment.

**Figure 9:**
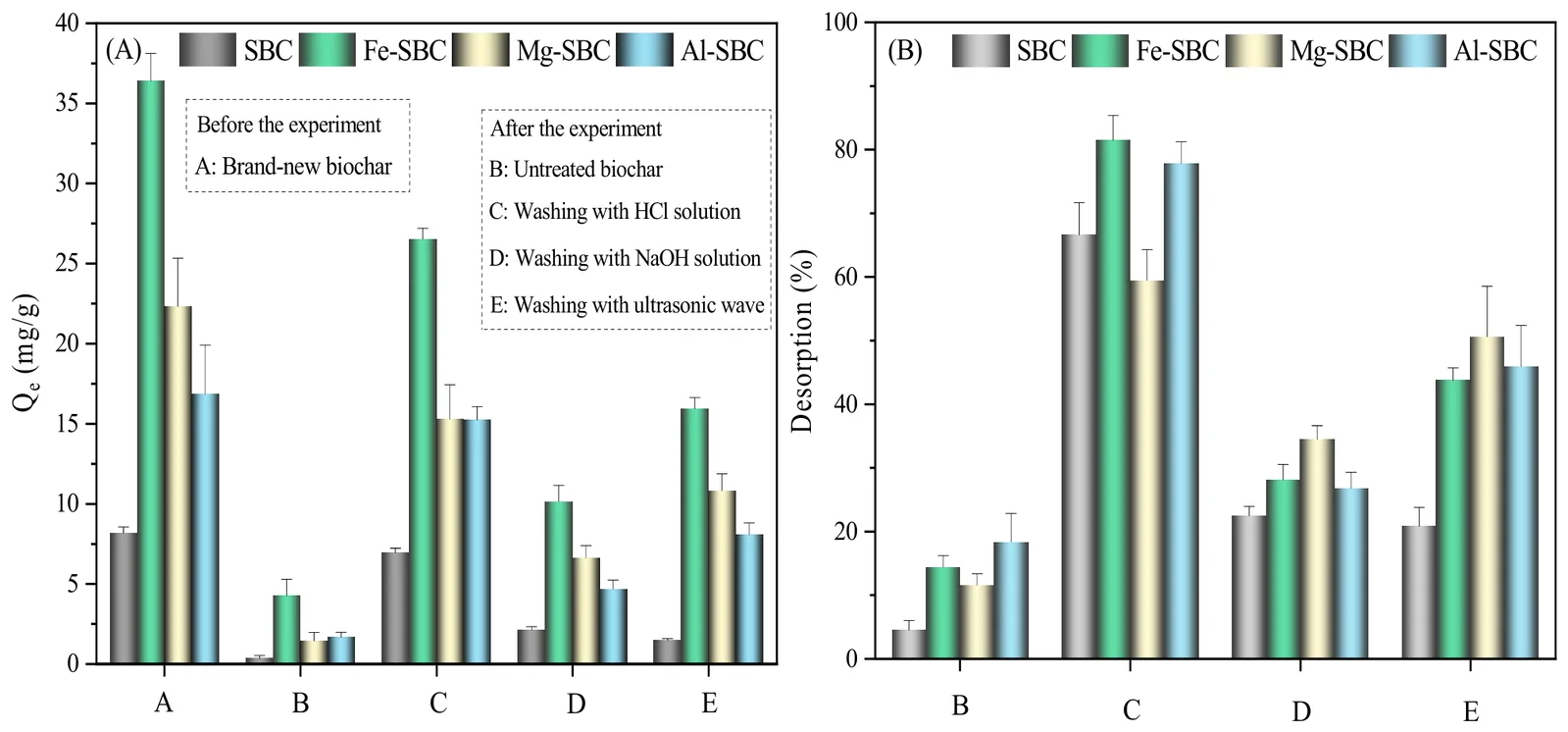
Adsorbing capacities (**A**) and desorption efficiency (**B**) of different biochars.

In addition to environmental safety, the recyclability of biochar is another important factor affecting its practical applicability. Figure 9 shows the regeneration performance of the recovered biochars from the biochar tubule. The results indicated that the HCl achieved the highest desorption efficiency (66.56–81.45%), significantly higher than NaOH (22.44–34.41%) and ultrasonic washing (20.77–50.57%), indicating HCl is an effective regeneration reagent. The superior performance of HCl also suggests that immobilized Cr mainly existed as Cr(III) [44], consistent with the proposed immobilization mechanisms. However, future studies should further investigate the leaching of metal species loaded onto the biochar under different regeneration treatments to better understand their effects on Cr adsorption performance.

#### 2.6.2. Techno-Economic Evaluation

A preliminary techno-economic evaluation was conducted to assess the practical applicability of biochar tubules. Detailed cost calculations are provided in the Supplementary Material. The laboratory-scale preparation costs of Fe-SBC, Mg-SBC, and Al-SBC were estimated to be 48.21, 48.90, and 73.15 RMB/kg, respectively. Based on their Cr removal capacities, the corresponding Cr removal costs were calculated as 6.85, 15.92, and 27.50 RMB/g, respectively. Considering the balance between preparation cost and removal performance, Fe-SBC was identified as the most cost-effective material. Moreover, the biochar tubule recycling potential could further reduce its practical application cost, indicating the promising economic feasibility of the biochar tubule technology for Cr-contaminated soil remediation.

#### 2.6.3. Comprehensive Comparison and Application Prospects

Compared to conventional immobilization methods involving thorough biochar–soil mixing, the biochar tubule demonstrated reduced effectiveness in delaying HM breakthrough and decreasing their release amounts. For example, Wang et al. [45] mixed biochar with As-contaminated soil and found that As passed through the soil column when the leaching volume reached 1350 mL, whereas breakthrough (peak concentration) occurred at 300 mL in this study. ChenDai [9] found that Fe(III)-modified biochar effectively reduced the Cr leaching amount by 81% in a contaminated soil column with the Cr(VI) content at 6622 mg/kg, compared to a reduction of 71.40% observed in this study (Cr content: 1424 mg/k). Zhao et al. [46] considered that mixing biochar with soil can introduce more biochar fragmentation to form a denser matrix to delay the passage of HMs.

The immobilization effects observed in this study were less effective than those of conventional immobilization methods; however, they still achieved relatively satisfactory results in immobilizing Cr(VI). Additionally, applying biochar tubule to immobilize Cr(VI) only requires inserting it into contaminated soil rather than the labor-intensive mixing operations. This method offers simple and flexible application, proving particularly effective for slightly Cr(VI)-contaminated soils, and warrants promotion in engineering applications.

## 3. Materials and Methods

### 3.1. Chemicals and Raw Materials

All chemicals were analytical grade or superior grade. Walnut shells and sludge were collected from a local village and a wastewater treatment plant in Chengdu, respectively. The raw materials were dried, ground, and sieved (100 mesh) prior to use. Soil samples (0–40 cm) were collected from uncultivated land using the quincunx sampling method. The basic physicochemical properties of the soil and sludge are summarized in Table S5.

### 3.2. Preparation of Modified Biochar and Contaminated Soil

Modified biochars were prepared via a dual-modification strategy combining metal salt impregnation and co-pyrolysis under oxygen-limited conditions. Briefly, sludge and walnut shell powder were mixed at a mass ratio of 1:1 and impregnated in 2M FeCl_3_, MgCl_2_ or AlCl_3_ solutions (1:2 *w*/*v*) for 24 h [7]. The mixtures were then dried (105 °C), ground (100 mesh), and placed in crucibles tightly covered with aluminum foil (oxygen-limited environment) before pyrolysis at 500 °C for 2 h. The obtained biochars were designated as Fe-SBC, Mg-SBC, and Al-SBC. Contaminated soil was prepared by spiking with a potassium dichromate (K_2_Cr_2_O_7_) to achieve Cr concentrations of 250 mg/kg and 1300 mg/kg, corresponding to the risk screening values (RSVs) and risk intervention values (RIVs) defined in the Chinese standard (GB 15618-2018) [47].

### 3.3. Characterization of Biochar

The S_BET_, pore size distribution, micromorphology, elemental composition, functional groups, and crystal structure of the biochars were characterized using various analytical techniques. These included Brunauer–Emmett–Teller (BET) surface area analysis (ASAP 2460, Micromeritics, Norcross, GA, USA), scanning electron microscopy with energy-dispersive spectroscopy (SEM-EDS) (Nova Nano, SEM450, FEI, Hillsboro, OR, USA), an elemental analyzer (Vario EL III, Hanau, Germany), Fourier transform infrared spectroscopy (FTIR) (Nicolet iS 20, Thermo Scientific, Waltham, MA, USA), and X-ray diffraction (XRD) (Ultima IV, Rigaku Corporation, Akishima-shi, Japan). The fundamental physicochemical properties of the biochar, soil, and sludge are summarized in the Supporting Materials (Sections S1.1–S1.3).

### 3.4. Experiment Design

#### 3.4.1. Column Experiment

**Figure 10:**
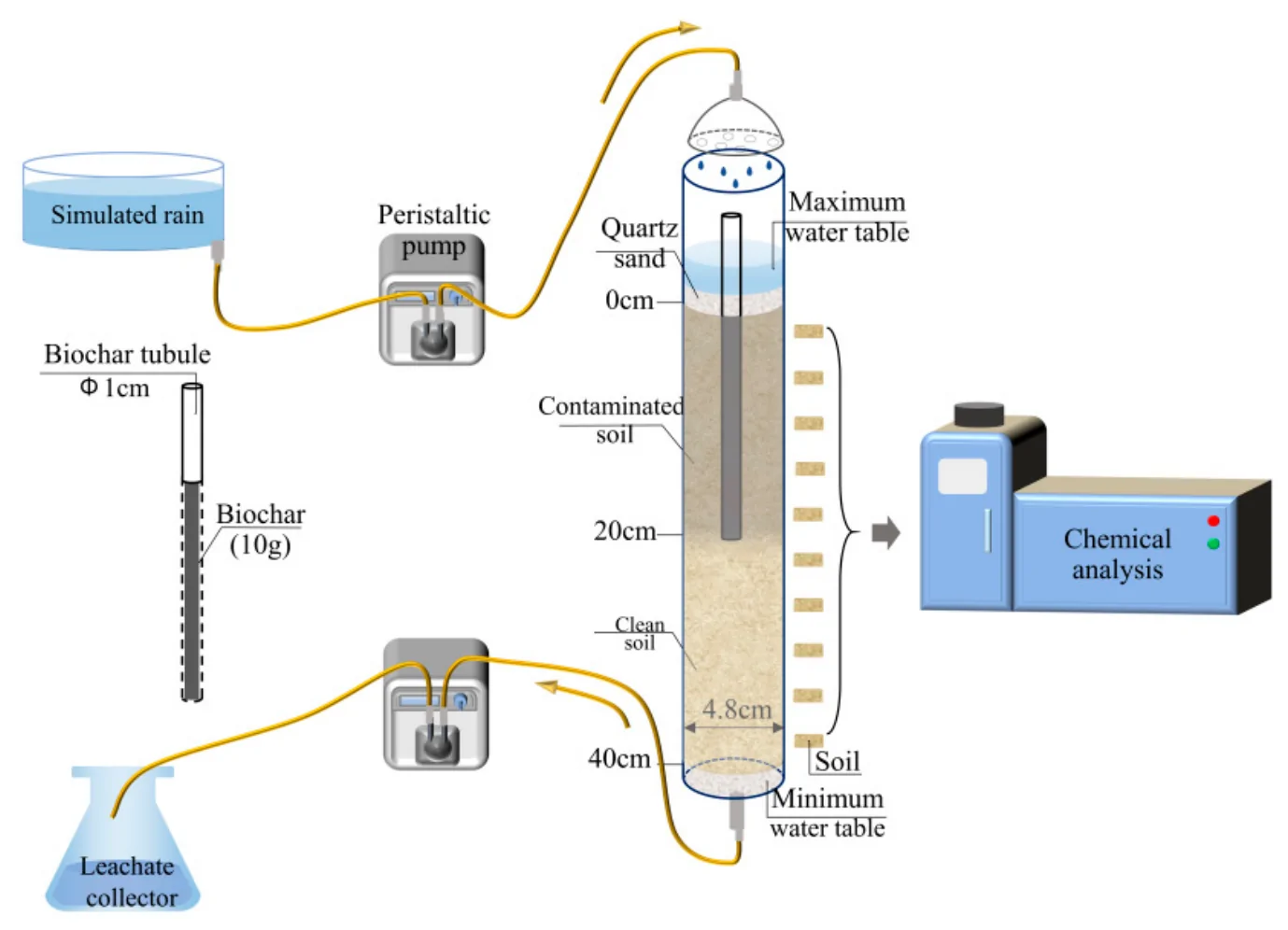
Schematic diagram of the experimental setup.

Column experiments were conducted to evaluate the performance of biochar tubules for Cr immobilization under GTF conditions. Before packing, the column inner walls were coated with a thin layer of Vaseline to minimize preferential flow. Subsequently, the columns were uniformly packed with either uncontaminated or Cr-spiked soil. A porous tubule loaded with one of the biochar types (Fe-SBC, Mg-SBC, Al-SBC, or SBC) was then inserted into the center of each soil column, while quartz sand was used instead of biochar in the control (CK) group. Quartz sand was added to ensure steady infiltration, while a peristaltic pump precisely controlled the GTF process (Figure 10). The biochar dosage was set at 2.2% (*w*/*w*), which determined the diameters of both the column (4.8 cm) and tubule (1.0 cm). The column height was 40 cm, representing the precipitation-sensitive soil zone [48]. For comparison, control experiments were conducted under continuous drying (unsaturated) and flooding (saturated) conditions alongside the GTF treatment (variably saturated).

Based on local rainfall (964 mm) and an assumed 30% surface runoff loss [49], the rain volume was calculated as 1.2 L (Equation (1)). The pH value and ionic composition of the rainwater are provided in the Supporting Materials, Section S2. The leachate infiltration rate was set to 0.28 mL/min, corresponding to 2.3 × 10^−4^ cm/s, which is comparable to typical field infiltration velocities reported in previous studies [50]. The experiment consisted of four GTF cycles. At the start of each GTF cycle, rain was leached within 60 min to reach the maximum water table. Precipitation was paused until leachate depletion, then resumed. When the rain reached the maximum water table again, the precipitation was reduced to maintain a constant water table. As the rain dried up, the water table decreased to its minimum level, completing the single GTF process. The soil columns were then allowed to stand for 72 h before beginning the next GTF process, continuing this process until the entire experiment was completed. During the experiment, leachate samples were collected every 0.1 L to measure total Cr. After completion, biochar tubules were retrieved, and soil samples were collected at 10 equal intervals along the column to determine the total Cr, labile fractions and Cr speciation.

(5) $$V=0.964\;m\times 0.7\times 3.14\times {\left(0.048\right)}^{2}\times 1/4\times 10^{3}=1.2\;L$$

#### 3.4.2. Adsorption–Desorption Experiment

The adsorption experiments were conducted to evaluate the Cr adsorption capacity of the biochars. Briefly, 0.1 g of biochar was added to 50 mL of K_2_Cr_2_O_7_ or CrCl_3_ solution (100 mg/L) and shaken for 24 h; the Cr concentration was determined using ICP-OES (700T, BOVEI, Suzhou, China). Desorption experiments were conducted to assess the regeneration potential. Briefly, spent biochar (0.50 g) recovered from the tubules was washed with deionized water (ultrasonic), 0.5 M HCl, or 0.5 M NaOH (200 mL) for 12 h, followed by filtration and rinsing. The regeneration performance was assessed via a Cr(VI) adsorption experiment. The adsorption–desorption procedure was repeated for three cycles, and the Cr concentrations were determined after the final cycle using ICP-OES.

### 3.5. Analytical Methods and Data Processing

Total Cr and other HMs in the soil, biochar, and sludge were determined by ICP-OES after mixed-acid digestion, while the leachate samples were analyzed immediately. Soil Cr speciations were analyzed using Tessier’s extraction procedure [51], which divides Cr into five fractions: exchangeable (F1), carbonate-bound (F2), Fe-Mn oxide-bound (F3), organic matter-bound (F4), and residual (F5) fractions, with their stability generally increasing from F1 to F5. Commonly, F1 and F2 were defined as labile fractions, whereas F3–F5 were considered relatively stable fractions. Detailed analysis procedures are provided in the Supporting Materials (Section S1.2). Cr(VI) in the biochar was extracted with 0.1 M NaOH and quantified by a UV–vis spectrophotometer (Metash, UV-5100, China, Shanghai) at 540 nm.

All data are presented from the average values. Significant differences (*p* < 0.05) among treatments were assessed using Duncan’s test. The XRD patterns were analyzed using Jade 6.5, and data processing was performed with Excel 2016 and Origin 2022.

## 4. Conclusions

This study systematically evaluated the Cr immobilization performance, underlying mechanisms, and engineering potential of different SBC tubules under GTF conditions using soil column experiments. The characterization results demonstrated that modification effectively improved the physicochemical properties of the biochar, including the S_BET_, pore structure, and surface chemical properties. Column experiments revealed that the modified biochars significantly inhibited Cr leaching, while enhancing Cr retention in the soil and biochar, and promoting the transformation of Cr into more stable fractions. Among all treatments, Fe-SBC exhibited the best immobilization performance, followed by Mg-SBC, Al-SBC, and SBC. Specifically, compared with CK, Fe-SBC reduced cumulative Cr release by 97.66% (RSV) and 71.40% (RIV) in the soils, while increasing the residual Cr amounts by 155.68% and 257.42%, respectively. Moreover, the retained Cr was predominantly present in stable fractions, accounting for 97.46% (RSV) and 88.02% (RIV) in the soil. In addition, 7.05% and 9.05% of the Cr total amounts were sequestered within the biochar tubule, respectively. Mechanistic analysis revealed that Cr(VI) was transported into the biochar tubules through preferential flow induced by the tubular, where it was synergistically immobilized via adsorption, reduction, precipitation, and complexation. Among these mechanisms, redox reactions played a dominant role (67.37–98.87%) in immobilizing Cr(VI). Engineering evaluation demonstrated that the modified biochar exhibited low environmental risks, with a Cr removal cost (Fe-SBC) of approximately 6.85 RMB/g. Meanwhile, the biochar tubule system showed advantages in operational simplicity, recoverability, and reduction in long-term environmental risks. This study provides a promising approach for sludge valorization and Cr-contaminated soil remediation under fluctuating groundwater conditions.

## Figures and Tables

**Table 1:** Comparison of Cr(VI) and Cr (III) adsorption capacities of different biochars.

Biochars	Pyrolysis Conditions	Initial Concentration (mg/L)	Sorbent Dose (g/L)	Adsorption Capacity (mg/g)	Ref.
SBC	500 °C, 2 h	Cr(VI): 100	2.0	8.13	Present work
Fe-SBC				36.37	
Mg-SBC				22.3	
Al-SBC				16.82	
Sugarcane residue biochar	550 °C, 1 h	Cr(VI): 100	1.0	9.21	[19]
FeCl_3_-modified straw biochar	500 °C, 2 h	Cr(VI): 100	5.0	34.10	[20]
ZVI-modified sludge biochar	220 °C, 2 h	Cr(VI): 500	1.0	73.69	[21]
SBC	500 °C, 2 h	Cr (III): 100	2.0	24.12	Present work
Fe-SBC				18.10	
Mg-SBC				51.17	
Al-SBC				28.13	
Poultry manure biochar	450 °C, 3 h	Cr (III): 50	5.0	33.94	[22]
Tomato stems biochar	250 °C, 1 h	Cr (III): 300	1.0	62.20	[23]
Macroalga biochar	800 °C, 1 h	Cr (III): 300	1.0	163.90	[24]

**Table 2:** Cr mass balance in different components of soil columns after biochar treatment.

Biochar	Initial Cr Mass (mg)	Final Cr Mass Distribution (mg)					Mass Balance Error (%)
		Upper Soil	Biochar	Lower Soil (20–40 cm)	Leachate	Total Recovered Cr	
CK	RSV (218.67)	84.30	0	53.81	67.04	205.15	6.18
SBC		85.69	7.14	61.03	56.31	210.17	3.89
Fe-SBC		148.77	16.38	70.06	1.57	236.78	8.28
Mg-SBC		102.98	11.91	65.36	46.50	226.75	3.70
Al-SBC		99.19	9.60	64.87	50.29	223.95	2.40
CK	RIV (738.16)	128.25	0	84.72	553.49	766.46	3.84
SBC		147.73	14.36	104.79	509.90	776.78	5.23
Fe-SBC		307.71	70.40	240.70	158.31	777.11	5.28
Mg-SBC		182.27	30.73	116.04	415.33	744.38	1.25
Al-SBC		173.73	26.56	98.91	442.55	741.74	0.90

**Table 3:** Changes in HM concentrations in biochar before and after the experiment, mg/kg.

Samples		SBC	Fe-SBC	Mg-SBC	Al-SBC
RSV	Cu	−1.27 ^a^	−18.39	−23.64	−22.19
	Pb	−8.19	−28.05	−19.65	−20.16
	Cd	0.19 ^b^	0.01	0.03	0.01
	Zn	12.48	−12.05	−21.82	−13.41
	Ni	26.82	3.25	2.52	3.31
RIV	Cu	1.56	−14.55	−18.64	−15.30
	Pb	−1.73	−45.07	−33.98	−24.29
	Cd	0.15	0.02	0.05	0.03
	Zn	21.09	−2.30	−4.09	−3.36
	Ni	44.03	10.28	8.07	11.01

Note: (^a^) Negative values indicate adsorption of HMs by biochar; (^b^) positive values indicate release from biochar to soil.

## Data Availability

The original contributions presented in this study are included in the article.

## Supplementary Materials

The following supporting information can be downloaded at: https://www.mdpi.com/article/10.3390/molecules31173068/s1. Figure S1. SEM images of different biochars; Figure S2. SEM and ESD images after the immobilization effect; Table S1. Physical and chemical properties of different biochars; Table S2. Elemental composition of sewage sludge (SS), walnut shell (WS), and biochars; Table S3. The Fe, Mg, Al concentrations in soil Before and after the experiment (mg/kg); Table S4. The potential ecological risk index of HMs in soil; Table S5. Basic properties of sludge and soils [50,51,52,53,54,55].
