# Supplementary material for: Hexavalent Chromium Immobilization in Soil by Sludge Biochar Tubules Under Fluctuating Groundwater: Performance, Mechanisms and Engineering Potential
Source: Molecules. 2026 Aug 31;31(17):3068. doi: 10.3390/molecules31173068 (PMC13567344; doi:10.3390/molecules31173068)
Supplement: Supplementary file 1 [file molecules-31-03068-s001.zip › molecules-4492978-supplementary.pdf]

Supporting Information for

Hexavalent Chromium Immobilization in Soil by Sludge Biochar Tubules under Fluctuating Groundwater: Performance, Mechanisms and Engineering Potential

Lin Chen [a, b] ,Yinger Deng [b, †], Yurou Hu[b].

[a] Key Laboratory of Synergetic Control and Joint Remediation for Soil & Water Pollution, Ministry of Ecology and Environment, Chengdu University of Technology, Chengdu, 610059, China

[b] College of Environment and Civil Engineering, Chengdu University of Technology, Chengdu 610059, China

† Corresponding author: Yinger Deng.

E-mail address:

dengyinge@mail.cdut.edu.cn (Yinger Deng)

Postal address: 1#, Dongsanlu, Erxianqiao, Chengdu 610059, Sichuan province, the People's Republic of China. Chengdu University of Technology.

E-mail address:

2022010108@stu.cdut.edu.cn (Lin Chen)

2019010103@stu.cdut.edu.cn (Yurou Hu)

S1. Characterizations

S1.1. Biochar

The yield was determined using the gravimetric method. The pH and EC values were measured with a pH meter (PHS-3C, Fangzhou Technology, China) and an EC meter (DDS-309, Fangzhou Technology, China) after shaking for 30 min (pH, 1:20; EC, 1:100, w/v). The total Cr and other HMs contents were analyzed using ICP-OES (700T, BOVEI, China) following mixed acid digestion (HNO_3: $HClO_4$: HF=5:5:2, v/v) [52].

S1.2. Soil

The pH and EC values were measured by a pH meter and an EC meter(pH, 1:2.5; EC, 1:5, w/v). The moisture content and bulk density were determined using the gravimetric method. The total Cr content was analyzed by ICP-OES following mixed acid digestion, as specified in the standard method of China (HJ 832-2017). The Cr chemical fractions were analyzed following the Tessier five-step sequential extraction procedure[51]. In Brief, the F1 fraction was extracted using $MgCl_2$ (8 ml, 1.0 M) at pH 7, followed by shaking for 60 min at 25 °C. The F2 fraction was extracted using NaAc (8 ml, 1.0 M) at pH

5, followed by shaking for 300 min at 25 °C. The F3 fraction was extracted using $NH_2OH·HCl$ (20 ml, 0.04 M), followed by shaking for 360 min at 96 °C. The F4 fraction was extracted using HNO_3 (3 ml, 0.02 M) and H_2O_2 (5 ml, 30%) at pH 2, followed by shaking for 120 min at 85 °C, and then further extracted using H_2O_2 (3 ml, 30%) with shaking for 180 min at 85 °C, The F5 fraction was extracted using the same procedure as that used for total Cr. The concentrations in F1-F5 fractions were measured using ICP-OES.

S1.3. Sludge

The moisture contents were measured by the gravimetric method after drying at 105 ℃. pH values were measured with a pH meter (1:10, w/v), shaking for 240 min. The total Cr content was measured using ICP-OES following mixed acid digestion in accordance with soil digestion procedures.

S2. Simulated Rain

The pH value of simulated rain was set as weakly acidic (pH=6.36), based on a report by the Chengdu Municipal Bureau of Ecological Environment in 2023 (Sichuan Province, China). The primary ions composition was determined according to the characteristics of rainwater in Chengdu, containing NH_4^+ (11.78 mg/L), K^+ (1.43 mg/L), Na^+ (0.24 mg/L), Ca^{2+} (19.89 mg/L), Mg^{2+} (3.03 mg/L), SO_4^{2-} (24.29 mg/L), NO_3^- (9.16 mg/L) and Cl^- (0.24 mg/L) [50].

S3. Tables

Table S1. Physical and chemical properties of different biochars.

Samples		SBC	Fe-SBC	Mg-SBC	Al-SBC
Surface area (m^2/g)		6.21	15.57	34.03	20.85
Total pore volume (cm^3/g)		0.047	0.044	0.115	0.040
Average pore diameter (nm)		31.24	11.22	13.50	7.00
Yield (%)		55.49±0.33	63.79±0.76	40.99±0.39	46.83±0.25
pH		7.93±0.7	2.10±0.02	9.79±0.03	4.36±0.06
EC (mS/cm)		0.10±0.02	3.84±0.04	1.83±0.10	0.43±0.04
Total Cr before experiment (mg/kg)		493.37±7.36	243.01±35.22	253.46±25.17	240.28±24.30
Total Cr after experiment (mg/kg)	RSV	713.95±34.67	1637.54±38.23	1190.53±55.54	960.33±3.40
Total Cr after experiment (mg/kg)	RIV	1435.60±28.56	7039.75±89.08	3072.97±113.09	2655.54±63.31

Table S2. Elemental composition of sewage sludge (SS), walnut shell (WS), and biochars.

Samples	Elemental Composition（%）					Atomic ratio	
	C	H	N	S	O[a]	H/C	O/C
SS	39.71	6.32	5.87	1.08	8.29	1.91	0.16
WS [53]	47.8	6.60	0.22	0.01	45.2	1.64	0.7
SBC	22.69	1.04	2.42	2.17	1.96	0.55	0.06
Fe-SBC	30.39	0.89	1.03	1.28	13.65	0.35	0.34
Mg-SBC	33.26	0.95	1.01	0.97	13.79	0.34	0.31
Al-SBC	31.07	0.99	1.06	1.18	9.55	0.38	0.23

a: O（%）=100-(C+H+N+S+Ash).

Table S3. The Fe, Mg, Al concentrations in soil Before and after the experiment (mg/kg)

Groups	Before the experiment			After the experiment (RSV soil)		
	Fe	Mg	Al	Fe	Mg	Al
Fe-SBC				2.30×10^4	4.63×10^3	4.67×10^4
Mg-SBC	2.03×10^4	4.61×10^3	4.66×10^4	2.10×10^4	4.82×10^3	4.68×10^4
Al-SBC				2.12×10^4	4.65×10^3	4.79×10^4

Table S4. The potential ecological risk index of HMs in soil

Biochar		E_r^i						RI	Risk level
		Cd	Zn	Pb	Ni	Cu	Risk level		
RSV	CK	ND[a]	1.38	9.54	10.14	6.76	Slight	27.81	Slight
	SBC	48.68	1.40	9.19	11.07	6.65	Moderate/Cd	73.06	Slight
	Fe-SBC	ND	1.29	6.51	11.60	6.52	Slight	25.92	Slight
	Mg-SBC	ND	1.25	5.27	11.81	5.95	Slight	24.29	Slight
	Al-SBC	ND	1.30	6.83	12.01	6.65	Slight	26.80	Slight
RIV	CK	ND	1.38	9.25	10.55	7.20	Slight	28.39	Slight
	SBC	44.76	1.43	9.34	12.73	7.13	Moderate/Cd	79.31	Slight
	Fe-SBC	ND	1.33	6.03	11.13	6.12	Slight	24.61	Slight
	Mg-SBC	ND	1.32	6.94	11.76	6.47	Slight	26.50	Slight
	Al-SBC	ND	1.36	7.18	11.53	6.64	Slight	26.71	Slight

Note：Risk assessment was conducted following the method of Lu, Yang [54].

Table S5. Basic properties of sludge and soils.

Properties \ Materials	Sludge	Raw soil	Artificial contaminated soil	
			RSV	RIV
Bulk density (g/cm^3)	/[a]	1.36±0.21	1.36±0.21	1.36±0.21
Moisture (%)	72.15±0.31	17.86±0.24	16.57±0.14	17.04±0.17
pH	5.76±0.07	8.37±0.02	8.03±0.04	7.69±0.09
EC (mS/cm)	/	0.15±0.01	0.24±0.02	0.68±0.04
Total Cr (mg/kg)	304±12.59	85.87±2.96	311.66±5.41	1424.22±21.64
F1 content of Cr (mg/kg)	/	ND	73.52±3.01	751.59±28.76
F2 content of Cr (mg/kg)	/	0.18±0.03	27.48±1.17	170.22±6.32
As (mg/kg)	128.61±12.67	4.66±0.37	/	/
Pb (mg/kg)	82.78±34.92	34.36±10.15		
Cd (mg/kg)	4.30±0.27	ND		
Ni (mg/kg)	346.98±17.38	45.54±1.42		
Location	104º07′08″ E, 30º39′51″ N,	104º9′5″ E, 30º40′56″ N		

[a]: Not tested.

ND: not detected

S4. Figures

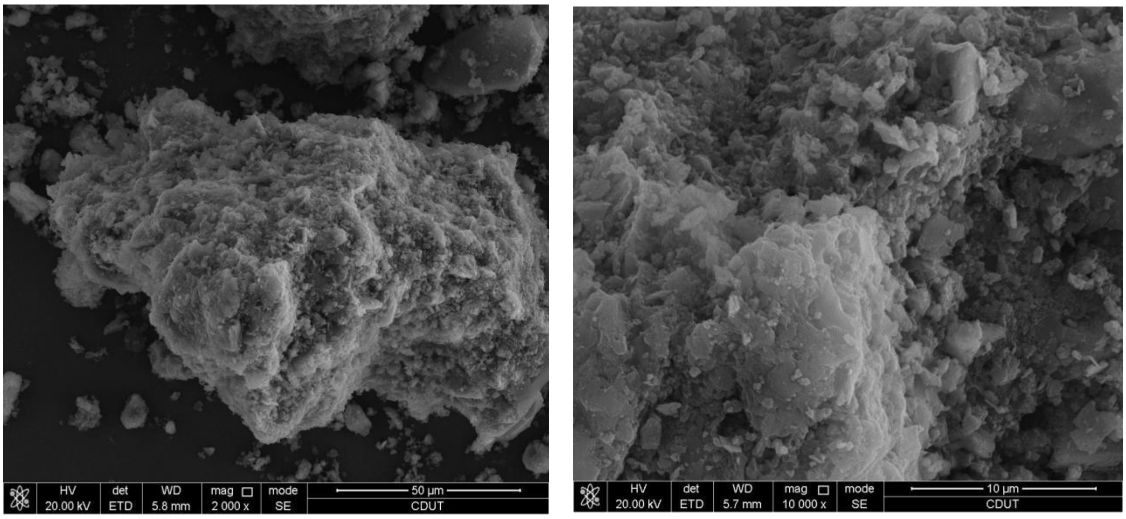

(A) SBC

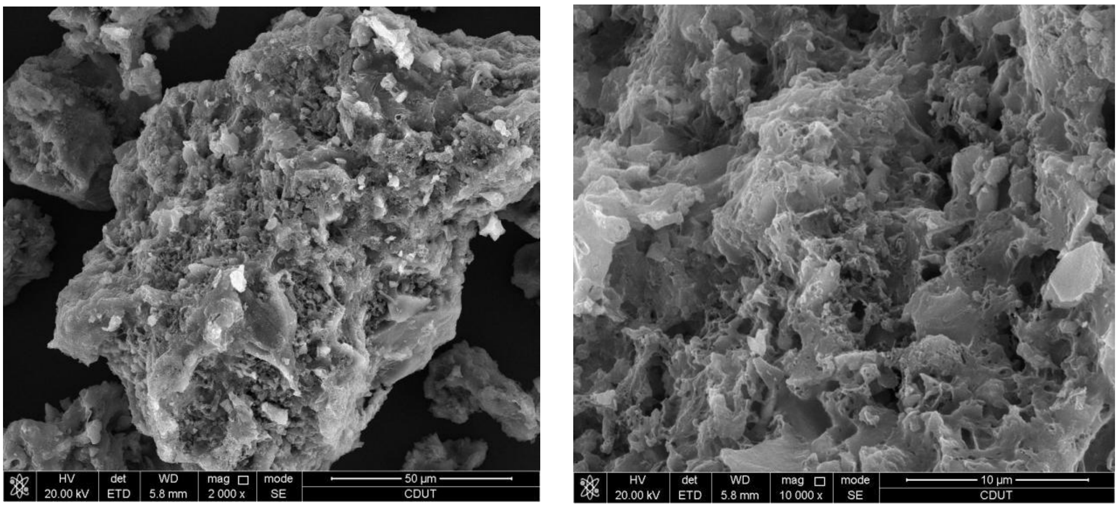

(B) Fe-SBC

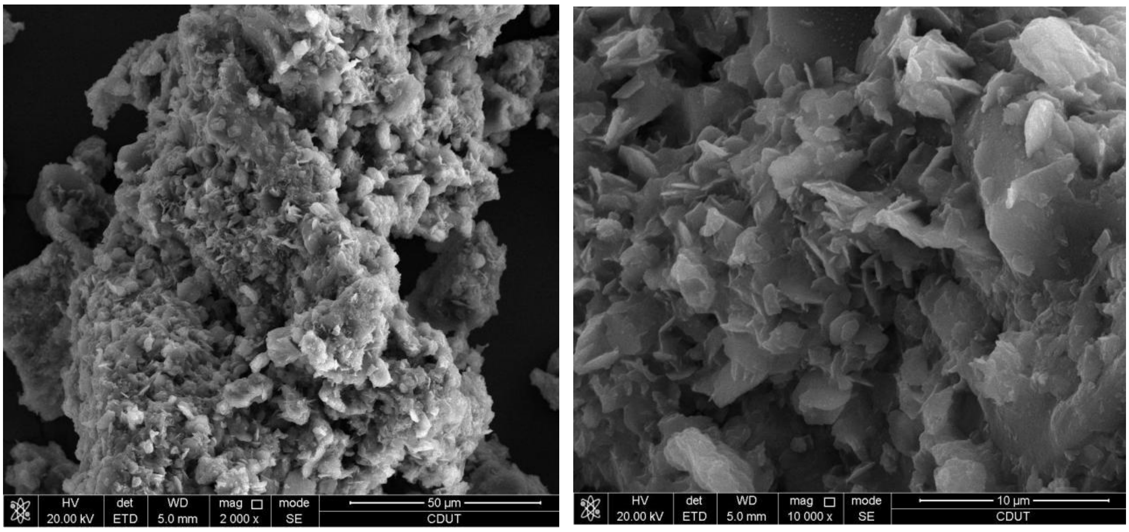

(C) Mg-SBC

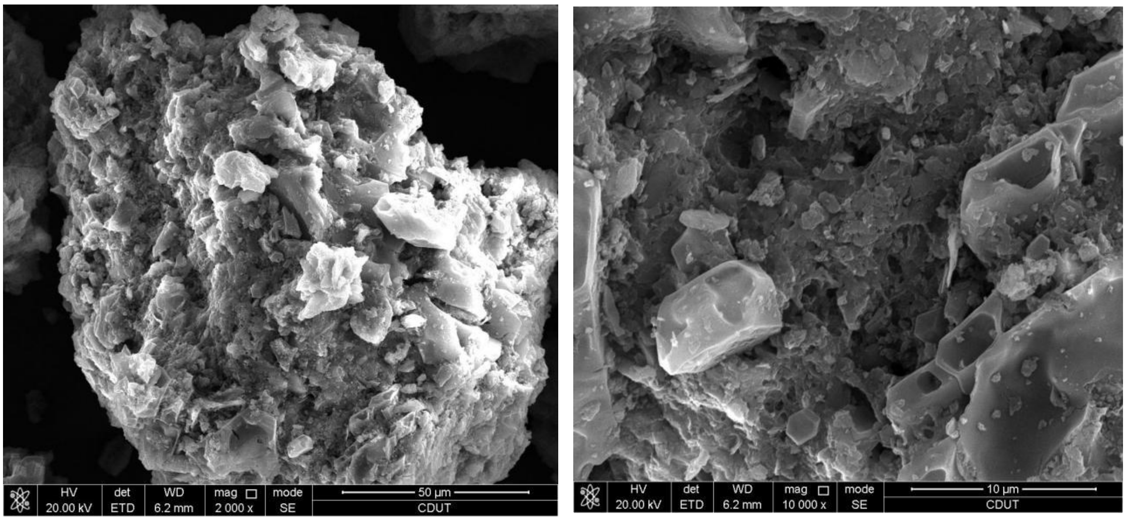

(D) Al-SBC

Figure S1. SEM images of different biochars.

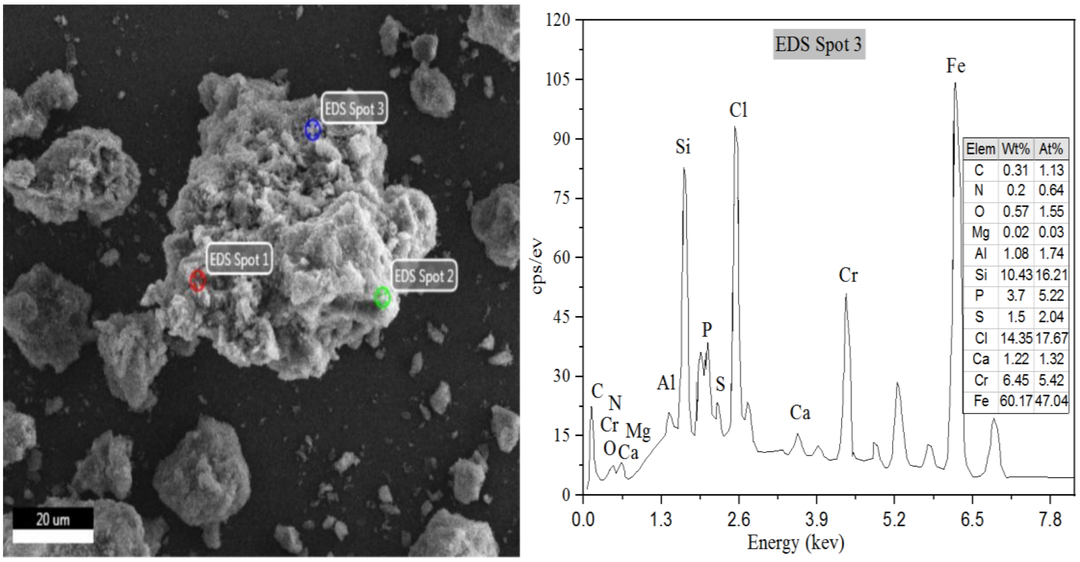

Figure S2. SEM and ESD images after the immobilization effect.

S5. Cost Estimation of Biochar Preparation

The cost of biochar preparation was estimated by considering material costs, electricity consumption, labor, and consumable expenses. The main raw materials for biochar production were sludge and walnut shell. The sludge was obtained from a wastewater treatment plant with negligible cost. Walnut shell powder was purchased from online suppliers at approximately 10 RMB/kg. The modification reagents, including $FeCl_3$, $MgCl_2$, and $AlCl_3$, cost approximately 64, 62, and 92 RMB/kg, respectively. Based on the raw material ratios and biochar yield (Eq. S1), the material costs for producing modified biochars were estimated to be 40.38, 41.00, and 63.05 RMB/kg, respectively.

$$F = [10 \times 0.5MS + 0 \times 0.5SS + 64 \times (nM\gamma \times 0.001)] \times \frac{1}{Y_B} \qquad \text{(S1)}$$

where F represents the material cost of biochar, (RMB/kg); $0.5MS$ and $0.5SS$ represent the amounts of walnut shell and sludge (kg), respectively; n represents the molar concentration (mol/L); M represents the molar mass (g/mol); γ represents the solid-to-liquid ratio; and Y_B represents the biochar yield.

The electricity cost mainly involved drying, grinding, and pyrolysis processes during biochar preparation. According to our previous study [55], the electricity consumption for producing biochar was approximately 3.45 RMB/kg. Moreover, the costs of consumables (e.g., aluminum foil, filter membranes, and containers) and labor were estimated as 10% of the total preparation cost. Therefore, the overall laboratory-scale preparation costs of the biochars were estimated to be approximately 48.21, 48.90, and 73.15 RMB/kg.
